# Current knowledge gaps in extracorporeal respiratory support

**DOI:** 10.1186/s40635-023-00563-x

**Published:** 2023-11-14

**Authors:** Tommaso Tonetti, Alberto Zanella, David Pérez-Torres, Giacomo Grasselli, V. Marco Ranieri

**Affiliations:** 1 Department of Medical and Surgical Sciences (DIMEC), Alma Mater Studiorum-University of Bologna, Bologna, Italy; 2 Anesthesiology and General Intensive Care Unit, IRCCS Azienda Ospedaliero-Universitaria di Bologna, Policlinico di S.Orsola, Bologna, Italy; 3 Department of Anesthesia, Critical Care and Emergency, Fondazione Istituto di Ricovero e Cura a Carattere Scientifico Ca’ Granda Ospedale Maggiore Policlinico, Via F. Sforza 35, 20122 Milan, Italy; 4 Department of Pathophysiology and Transplantation, University of Milan, Milan, Italy; 5 Servicio de Medicina Intensiva, Hospital Universitario Río Hortega, Gerencia Regional de Salud de Castilla y León (SACYL), Calle Dulzaina, 2, 47012 Valladolid, Spain

**Keywords:** V-V ECMO, Acute respiratory failure, Gas exchange, Lung protective ventilation

## Abstract

Extracorporeal life support (ECLS) for acute respiratory failure encompasses veno-venous extracorporeal membrane oxygenation (V-V ECMO) and extracorporeal carbon dioxide removal (ECCO_2_R). V-V ECMO is primarily used to treat severe acute respiratory distress syndrome (ARDS), characterized by life-threatening hypoxemia or ventilatory insufficiency with conventional protective settings. It employs an artificial lung with high blood flows, and allows improvement in gas exchange, correction of hypoxemia, and reduction of the workload on the native lung. On the other hand, ECCO_2_R focuses on carbon dioxide removal and ventilatory load reduction (“ultra-protective ventilation”) in moderate ARDS, or in avoiding pump failure in acute exacerbated chronic obstructive pulmonary disease. Clinical indications for V-V ECLS are tailored to individual patients, as there are no absolute contraindications. However, determining the ideal timing for initiating extracorporeal respiratory support remains uncertain. Current ECLS equipment faces issues like size and durability. Innovations include intravascular lung assist devices (ILADs) and pumpless devices, though they come with their own challenges. Efficient gas exchange relies on modern oxygenators using hollow fiber designs, but research is exploring microfluidic technology to improve oxygenator size, thrombogenicity, and blood flow capacity. Coagulation management during V-V ECLS is crucial due to common bleeding and thrombosis complications; indeed, anticoagulation strategies and monitoring systems require improvement, while surface coatings and new materials show promise. Moreover, pharmacokinetics during ECLS significantly impact antibiotic therapy, necessitating therapeutic drug monitoring for precise dosing. Managing native lung ventilation during V-V ECMO remains complex, requiring a careful balance between benefits and potential risks for spontaneously breathing patients. Moreover, weaning from V-V ECMO is recognized as an area of relevant uncertainty, requiring further research. In the last decade, the concept of Extracorporeal Organ Support (ECOS) for patients with multiple organ dysfunction has emerged, combining ECLS with other organ support therapies to provide a more holistic approach for critically ill patients. In this review, we aim at providing an in-depth overview of V-V ECMO and ECCO_2_R, addressing various aspects of their use, challenges, and potential future directions in research and development.

## Introduction: current available techniques and indications

Veno-venous extracorporeal membrane oxygenation (V-V ECMO) and extracorporeal carbon dioxide removal (ECCO_2_R) are extracorporeal life support (ECLS) systems, indicated in different severity degrees of acute respiratory failure [1].

The current main indication for V-V ECMO is severe acute respiratory distress syndrome (ARDS) with either life-threatening hypoxemia or inability to ventilate with protective settings [2].

**Fig. 1:**
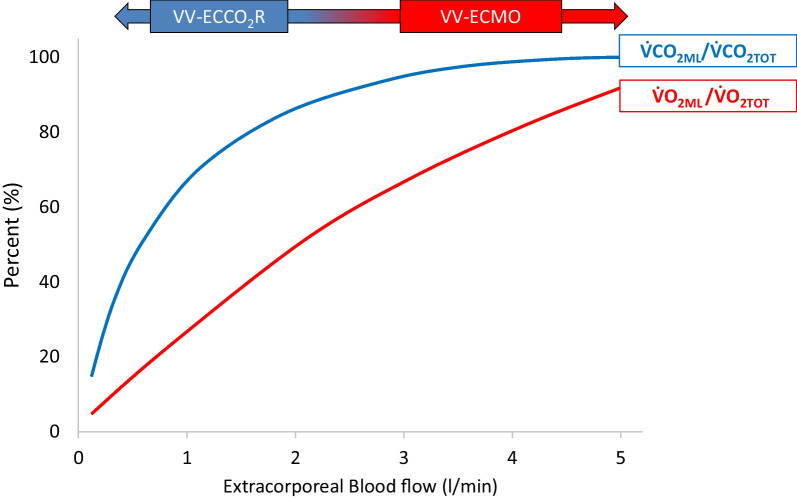
Extracorporeal oxygen delivery ($\dot{V}$ O_2ML_)/total oxygen consumption ($\dot{V}$ O_2TOT_) and extracorporeal carbon dioxide removal ($\dot{V}$ CO_2ML_)/total carbon dioxide production ($\dot{V}$ CO_2TOT_) as a function of extracorporeal blood flow (BF) at steady state in an adult patient. $\dot{V}$ O_2TOT_ = 250 mL/min and $\dot{V}$ CO_2TOT_ 200 mL/min. Veno-venous extracorporeal oxygenation support (V-V ECMO), veno-venous extracorporeal CO_2_ removal (V-V ECCO_2_R)

Under V-V ECMO, highly protective mechanical ventilation is obtained by reducing the ventilatory load (pressures, volumes and rate) on the native lung, whose gas exchange function is undertaken by an artificial (membrane) lung. The membrane is perfused by high blood flows (normally 4–6 L/min), and the “sweep gas” (with a variable concentration of oxygen) is forced through the membrane’s fibers, with efficient oxygen and carbon dioxide exchange [3]. The resulting oxygenated and de-carboxylated blood is then mixed in the right atrium with the venous return not passing the membrane lung, and finally flows through the patient’s native lung, which contributes (at a variable degree) to the final PaO_2_ and SaO_2_. However, the two most important determinants of oxygenation under V-V ECMO are the extracorporeal blood flow (Fig. 1) and the fraction of oxygen in the sweep gas (FmO_2_).

Differently from ECMO, ECCO_2_R uses lower blood flows, smaller circuits, membranes, and cannulas. As such, the main effect of this technique is to lower carbon dioxide, without any relevant effect on oxygenation (see Fig. 1). Indeed, the principal determinant of carbon dioxide removal is the amount of sweep gas flow. Accordingly, ECCO_2_R uses blood flows in the range of 300–1500 mL/min and sweep gas flows up to 8–10 L/min. Clinical indications for ECCO_2_R are still debated, but it is presently applied in severe exacerbations of chronic obstructive pulmonary disease (COPD) (with the aim of reducing the rate of intubation and duration of mechanical ventilation) and in moderate ARDS (to reduce the ventilatory load to allow “ultra-protective” ventilatory settings) [4, 5]. Combinations of ECCO_2_R with continuous renal replacement therapy (CRRT) have been recently described in patients with ARDS and acute kidney injury [6, 7].

In each section of this review, we will describe the current state of the art and, thereafter, the gaps in knowledge and potential research directions in the field of VV-ECLS.

## Clinical indications and contraindications for V-V ECMO

Current indications for V-V ECLS are summarized in Table 1. The two main “classical” indications for V-V ECMO are life-threatening hypoxemia and/or the inability to ventilate with protective ventilatory settings in patients with potentially reversible severe ARDS [8] or other forms of acute hypoxemic respiratory failure such as interstitial lung disease [9] and as a bridge to lung transplant [10]. Several prediction scores have been proposed to assist clinicians in properly evaluating candidates [11–13].

**Table 1.** Current indications for V-V ECLS

Technique	Indications
V-V ECMO	Severe ARDS with one of following, after optimization of MV [18]: • PaO_2_/FiO_2_ < 50 mmHg with FiO_2_ ≥ 0.8 for > 3 h • PaO_2_/FiO_2_ < 80 mmHg with FiO_2_ ≥ 0.8 for > 6 h • pH < 7.25 (with PaCO_2_ ≥ 60 mmHg) for > 6 h (with RR increased to 35 bpm)
V-V ECCO_2_R	• Moderate ARDS with P_plat_ > 30 cmH_2_O after optimization of MV [19] • Acute hypercapnic exacerbation of COPD, with two of following, after 2 h of NIV [20]: – arterial pH ≤ 7.25 – RR ≥ 30 bpm – use of accessory muscles or paradoxical abdominal movements
Combined CRRT-ECCO_2_R	Mild or moderate ARDS with AKI needing CRRT [21]

*ARDS* acute respiratory distress syndrome; *bpm*, breaths per minute; *FiO2* inspiratory fraction of oxygen; *NIV*, non-invasive ventilation; *PaCO2* arterial partial pressure of carbon dioxide; *PaO2* arterial partial pressure of oxygen; *Pplat* airway plateau pressure; *RR* respiratory rate; *COPD* chronic obstructive pulmonary disease; *CRRT* continuous renal replacement therapy; *AKI* acute kidney injury

Besides this oversimplified view, there are still many “gray areas” of possible use of ECMO. Indeed, the ELSO guidelines state no absolute contraindications for V-V ECMO, since each patient is considered as a candidate individually with regard to potential risks and benefits. Careful consideration is warranted in patients with severe comorbidities, older age [14, 15], multiorgan failure, intracranial bleeding, mechanical ventilation for > 7 days at high settings (FiO_2_ > 0.9, plateau pressure > 30 cmH_2_O), or major pharmacological immunosuppression (absolute neutrophil count < 400/mm^3^) [16]; indeed, these patients could probably have a poor prognosis despite successful V-V ECMO treatment. Moreover, the ideal timing to consider extracorporeal respiratory support after the initiation of mechanical ventilation remains unclear. Patients on mechanical ventilation for 7 days or longer were excluded in all recent trials, with prolonged mechanical ventilation being an independent predictor of in-hospital mortality [17].

As for V-V ECCO_2_R, relevant clinical indications could be moderate ARDS with low static compliance and acute hypercapnic exacerbations of COPD. These are both covered in depth in another section of this review entitled ‘Low/medium-flow ECLS for protective lung ventilation’.

## Equipment and new technology

Modern equipment for V-V ECLS typically includes many different components [22]: (a) large-bore cannulas for drainage and return of the patient’s blood; (b) specialized tubing and connectors to conduct the blood; (c) a centrifugal pump that provides a continuous and consistent blood flow; (d) a membrane oxygenator; (e) a gas blender; (f) a heat exchanger; (g) sensors ensuring precise measurement of blood flow, pressures and oxygen levels; (h) a control and monitoring console, with a graphic display and human interface devices (usually knobs and buttons); and (i) alarm systems. The problems and shortcomings of current ECLS equipment may be summarized as follows: (a) excessive size; (b) lack of portability and mobility limitations; (c) external pump dependence; (d) lack of durability; and (e) lack of metabolic functions. Accordingly, research in the field is focusing on finding smaller and longer-running alternatives to present ECLS devices [23].

To address the problems related to excessive size and lack of portability, intravascular lungs assist devices (ILADs) have been developed since the late 1980s [24]. They are basically small implantable intravascular membrane oxygenators; both static and dynamic (i.e., impeller-driven) configurations have been developed and tested preclinically [25], with the aim of providing partial lung support. However, to date, its translation into the clinical world has proven impossible because of the lack of adequate blood and gas flows, pressure gradients and biocompatibility [23].

To address excessive dependence on external pumps, pumpless devices have been developed; they are based on an arterio-venous shunt and use the patient’s cardiac output as a prime mover. The most famous pumpless device, the interventional lung assist (iLA, NovaLung, Germany), has proven to be effective in removing CO_2_, with a limited effect on oxygenation [26]. Accordingly, the technique has been proposed both for allowing “ultra-protective” ventilation in ARDS [26] and as a bridge to lung transplantation in patients with ventilator-refractory hypercapnia [27]. However, pumpless systems have shown low oxygenation efficacy, difficult applicability (especially in patients with hemodynamic instability), risk of complications (especially limb ischemia), and low durability and have therefore been progressively abandoned [23].

The issue of circuits and oxygenators durability is deeply intertwined with that of coagulation management and surface coating; this topic will be more extensively discussed in the section ‘Coagulation management during V-V ECMO’.

## Gas exchange

As for other parts of equipment described above, presently used oxygenators are also very far from perfect. We will start by describing their structure and functioning and then move on to their shortcomings and possible future solutions.

Current oxygenators are based on the juxtaposition of multiple hollow fibers creating channels, with the most commonly used material being polymethylpentene (PMP). Compared to older materials, these fibers have better durability, cause less hemolysis, and cause less plasma leakage. In the oxygenator, O_2_ transfer is dependent on: (a) surface area and diffusion characteristics of the membrane; (b) O_2_ partial pressure (PO_2_) gradient between blood and sweep gas; and (c) blood flow. The surface area has been substantially increased by using hollow fibers and should not be a limiting factor in current adult oxygenators [28]. Increasing blood flow increases the number of fibers used and maximizes contact. However, increased blood flow can decrease transit time and reduce oxygenation. When 100% oxygen is used as sweep gas, the blood downstream the membrane lung has a PO_2_ up to 300–500 mmHg, with a maximized O_2_ content [29]. Further, the extracorporeal oxygen delivery at a given blood flow is highly dependent on the oxygen content of the blood entering the membrane lung. Almost 5 L/min of blood flow are required to provide an oxygen transfer comparable to the total body oxygen consumption of an adult patient (Fig. 1), assuming a hemoglobin saturation of 70% of the blood entering the membrane lung. Meanwhile, carbon dioxide is quickly transferred from the blood to the sweep gas according to the partial pressure gradient across the membrane. High sweep gas flows allow clearing an amount of CO_2_ comparable to 60% of the total CO_2_ production, while blood flows above 2–3 L/min may be required to remove an amount of CO_2_ comparable to the total CO_2_ production of an adult.

The shortcomings of presently used oxygenators fall into different domains: first, they are still voluminous and require relatively high priming volumes; second, they frequently induce blood cell trauma and hemolysis; and third, they tend to progressively lose efficiency over time due to coagulation and deposition of cells and fibers.

To address the shortcomings of currently available oxygenators, a significant amount of research is focused on microfluidic technology [23]. Systems utilizing this technology closely resemble the gas exchange interface of the native lung. They feature extremely thin channels, known as “microchannels”, that efficiently transport blood through the oxygenator’s gas-exchanging sections, while minimizing or even completely avoiding excessive shear stress, disturbed flow, and stasis [30]. Many different designs are presently under development and pre-clinical testing [30–37], but to date none of them have undergone clinical testing. One of the most relevant problems that needs to be solved prior to their clinical application is the relatively low blood flow that these oxygenators are presently able to sustain (i.e., 40–150 mL/min) [30]. Nevertheless, the technology is promising because it provides good gas exchange performance with lower thrombogenicity in a more compact size [23].

## Coagulation management during V-V ECMO

Bleeding and thrombosis are among the most common and dangerous complications during ECMO [38]. Although critical illness per se puts the patient into an altered coagulative state (sepsis, liver dysfunction, and disseminated intravascular coagulopathy being the main drivers), the most important causes of coagulopathy during ECMO are shear stress, turbulent flow, and exposure of blood to synthetic surfaces in the circuit [39]. Precise mechanisms have been described elsewhere and include on the one side activation of platelets and coagulation factors due to direct mechanical forces (promoting thrombosis), and on the other side configuration changes in von Willebrand factor and consumption of other factors due to high shear stress (promoting bleeding) [40].

A further challenge is represented by the partially inadequate diagnostic tests that are commonly used in patients on V-V ECMO. Activated clotting time (ACT) continues to be used for monitoring unfractionated heparin or direct thrombin inhibitors (DTIs), but concordance with other tests is often limited [41–43]. Activated partial thromboplastin time (aPTT) evaluates intrinsic and common pathways and is therefore used to monitor heparin and DTIs. Even so, aPTT is affected by levels of some endogenous factors (among others, fibrinogen and antithrombin), and therapeutic ranges can widely differ between different laboratories; thus, clinicians must individualize the approach [43]. Anti-factor Xa activity levels (anti-Xa) are increasingly used, as their correlation with heparin levels is higher than with aPTT. However, clot formation and firmness are not tested with this approach, and using it as a therapeutic target does not seem to improve outcomes [41, 44]. Viscoelastic tests (thromboelastography or rotational thromboelastometry) are based on the use of specific activators to evaluate the formation of clots in whole blood samples. While there is some evidence supporting their use for bleeding assessment and management, the use of viscoelastic tests for anticoagulation monitoring in ECLS is still a subject of debate [43].

As for the therapeutic regimens, unfractionated heparin (UFH) continues to be widely used in ECLS due to its short duration of effect, ease of use and monitoring, and possibility of reversal [38]. Nevertheless, some patients may develop dangerous complications, such as heparin-induced thrombocytopenia (HIT) and “heparin resistance”. The latter should be sought when ACT or aPTT targets cannot be reached with UFH infusion > 35,000 units/day [45]. Anti-Xa levels may be useful for diagnostic purposes in the setting of high heparin requirements to achieve the desired aPTT or ACT values [43]. Heparin effectiveness is also deeply influenced by antithrombin-III (AT-III) levels, and this factor is frequently supplemented during ECLS; however, despite its widespread use, only one RCT has been published to date, showing no effect of AT-III supplementation on heparin requirements or on the incidence of bleeding [46]. Some authors suggest that the use of low-molecular-weight heparins for anticoagulation in patients undergoing ECLS could be beneficial in terms of reduced thromboembolic and hemorrhagic events [47]. However, the evidence on survival benefits is still scarce and, more importantly, no RCTs have compared different anticoagulation strategies on ECLS to date [48]. Direct thrombin inhibitors (DTIs) are possible alternatives to UFH in cases of heparin resistance or HIT. Argatroban reversibly binds to and inhibits thrombin; having eminent hepatic elimination (a half-life of 40–50 min), its use should be preferred in cases of renal failure [49]. Bivalirudin reversibly inhibits thrombin, with an elimination half-life of 20–30 min, which can be variably prolonged in renal failure; it is widely used as a heparin alternative during intravascular interventions, but its use has occasionally been reported also during ECMO [49, 50].

Optimizing anticoagulation practices still appears to be a work-in-progress. To date, there is not any wide consensus among experts on the best therapeutic regimen, the most appropriate monitoring, or the management of thrombotic or bleeding complications. Accordingly, future research should focus on finding the best anticoagulation regimen, the best coagulation monitoring systems, and developing less thrombogenic materials [51, 52]. The most intuitive solution in this regard is to coat the contact surfaces with antithrombotic material [23]. Currently, the most commonly used coating is heparin, which has indeed led to a decrease in systemic anticoagulation needs [53]. However, more advanced coatings, such as nitric oxide-generating surfaces (for example, S-nitroso-N-acetyl-penicillamine and poly-carboxybetaine) are currently under investigation. Although promising in terms of thrombosis reduction, these approaches still need refinement before being applied in clinical trials [54, 55].

## Native lung ventilation during V-V ECMO

Mechanical ventilation can cause ventilator-induced lung injury (VILI) [56]. The classically recognized mechanisms of VILI include alveolar overdistension (barotrauma and volutrauma), cyclic alveolar collapse and reopening (atelectrauma), and consequent secondary inflammation (biotrauma). More recently, the unifying theory of ergotrauma has been proposed: the mechanical power (composed by pressures, volumes, respiratory rate, and flow) delivered to the lung should be the ‘*primum movens*’ of VILI [57]. Accordingly, “low power” ventilation (i.e., low tidal volume, low plateau pressure, and low respiratory rate) should reduce VILI but can possibly cause carbon dioxide retention and hypoxemia due to reduced ventilation [58].

V-V ECMO (and possibly ECCO_2_R) thus grants viable gas exchange while allowing protective (or even ultra-protective) settings. However, it should be noted that Gattinoni and Quintel recently proposed that the correct balance between lung rest and lung movement is difficult (if not impossible) to achieve [59, 60]. Indeed, during V-V ECMO, mechanical ventilation can often be required for two main reasons: (1) ECMO blood flow is not always sufficient to match the patient’s cardiac output (especially in hyperdynamic states), resulting in a substantial proportion of blood still passing exclusively through the native lung; (2) complete lung collapse due to hypoventilation may be detrimental for recovery from the underlying pathological mechanism [61].

Despite the lack of randomized controlled trials, there is a large body of observational evidence supporting the notion that protective ventilation during ECMO is generally associated with better outcomes. The reduction of plateau pressure seems to be particularly beneficial. In a retrospective series of more than sixty V-V ECMO patients, each cmH_2_O increase in plateau pressure was associated with a 14.4% decrease in the odds of achieving hospital survival [62]. On the contrary, the effect of positive end-expiratory pressure (PEEP) is more controversial. In a recent retrospective observational study, higher PEEP levels during the first 3 days of ECMO support were associated with lower mortality [63]. However, in another retrospective study considering PEEP values during the entire course of ECMO, every increase in PEEP (by 1 cmH_2_O) was associated with a 36.2% decrease in the odds of 30-day survival in multivariate analysis [64, 65].

In patients with severe ARDS, prone positioning has been associated with improved patient survival [66]. Prone positioning during ECMO is feasible, and it is possibly indicated in cases of severe hypoxemia (i.e., PaO_2_/FiO_2_ ratio < 70 mmHg), high plateau pressures (> 30 cmH_2_O despite protective settings), and difficulties in weaning from ECMO [61]. Although prone positioning may be safe if performed by properly trained personnel, there is evidence that it may entail significant risks to ECMO patients. Many severe adverse events have been reported, including cannula malfunction or malposition, accidental decannulation, unplanned extubation, bed sores, and dislodged arterial and central venous lines [67, 68].

Spontaneous breathing is usually not allowed during the early phases of severe ARDS: deep sedation and muscle relaxation are often needed to remove potentially injurious spontaneous effort and to allow protective mechanical ventilation [69]. Although early evidence showed a survival benefit with the use of cisatracurium in severe ARDS patients [70], these results were not confirmed in a later trial [71]. It is possible that adverse effects of paralysis, including respiratory muscle atrophy, which occurs as few as 18 h after the start of mechanical ventilation, may explain these results. The present vision is that restoration of respiratory muscle activity could possibly be helpful in decreasing or preventing such disuse myopathy [71].

However, it is not easy to find a satisfactory balance between the adverse effects of muscle relaxation and those of potentially injurious spontaneous breathing with effort, which warrants careful evaluation and continuous assessment. The removal of CO_2_ with an extracorporeal circuit can potentially control spontaneous breathing effort. Indeed, both in experimental settings and in observational clinical studies, an increase in CO_2_ removal by increasing gas and blood flow can modulate minute ventilation up to a condition of apnea [72]. Moreover, in a group of patients with chronic lung disease treated with ECMO as a bridge for lung transplantation, those spontaneously breathing demonstrated improved survival when compared to other bridging strategies [73].

Although allowing spontaneous breathing during ECMO seems physiological and could be tempting, especially because it may allow early mobilization and rehabilitation [74], there are still many uncertainties on indications and contraindications.

In an animal model of severe ARDS, Güldner and colleagues found that spontaneous breathing during V-V ECMO on the one side improved oxygenation and intrapulmonary shunt and redistributed ventilation towards dorsal areas, but on the other hand, ventilator-supported spontaneous breathing widely increased lung inflammation [75]. Moreover, in the early phases of severe ARDS, many patients show very high inspiratory efforts that are not adequately controlled even if PaCO_2_ is normalized by V-V ECMO [73]. Thus, allowing these patients to breathe spontaneously may be deleterious and less protective than controlled ventilation. Further studies in the field of neural control of ventilation are needed to fully understand and characterize this issue.

## Low/medium-flow ECLS for protective lung ventilation

As previously stated, ECCO_2_R is the designation for lower blood flow (usually less than 1000 mL/min) veno-venous ECLS devices. ECCO_2_R was initially proposed for the treatment of acute hypoxemic respiratory failure, but it was soon replaced by high-blood flow devices that allowed more effective oxygenation. Therefore, ECCO_2_R was “re-discovered” in more recent years for the treatment of hypercapnic respiratory insufficiency or for facilitating protective ventilation in ARDS patients when permissive hypercapnia was not tolerable [76, 77].

Hypercapnic exacerbations have a significant impact on survival (hospital mortality ~ 10%) and the quality of life of patients with COPD [78, 79]; moreover, they play a major role in increasing healthcare costs [80]. The standard of care remains non-invasive ventilation (NIV), which, however, is burdened by a high rate of failure, the need for intubation and a subsequent higher risk of death [78]. ECCO_2_R, even with very low blood flows (300–500 mL/min), has the potential to prevent NIV failure and therefore reduce intubation and mortality rates [81]. However, available evidence is limited to case series and matched cohort studies. Several randomized trials are planned [20], and they will hopefully provide more robust evidence on the use of ECCO_2_R in acute decompensations of COPD [82].

In ARDS, ECCO_2_R allows to dissociate oxygenation (managed by the native lung) from the removal of carbon dioxide, thus allowing ultra-protective ventilation strategies (V_T_ as low as 3–4 mL/Kg of PBW, plateau pressure < 30 cmH_2_O, reduction of respiratory rate, and minimization of mechanical power of ventilation). In observational studies, ultra-low V_T_ ventilation (3–4 mL/Kg of PBW) was associated with a significant decrease in inflammatory markers compared to standard low volume, low pressure ventilation [76, 83]. A preliminary RCT showed that V_T_ of 3 mL/Kg of PBW was easy and safe to implement with extracorporeal CO_2_ removal; when analyzing patients with PaO_2_/FiO_2_ < 150 mmHg, clinical outcome significantly improved in ECCO_2_R patients compared to controls [84]. A multicenter study showed that more than 80% of patients with moderate ARDS could achieve ultra-protective ventilation goals by using ECCO_2_R, and the incidence of severe adverse events was low (~ 2%) [19]. Interestingly, the efficacy and safety of ECCO_2_R were higher for devices that used blood flow in the range of 1000–1500 mL/min [85]. A more recent RCT, using lower-blood flow devices (up to 450 mL/min) in patients with acute hypoxemic respiratory failure, showed no difference in terms of mortality between ultra-protective ventilation with ECCO_2_R and standard protective ventilation, although ECCO_2_R patients had significantly lower ventilation-free days [86]. Other trials using higher blood flow devices are ongoing (clinicatrials.gov NCT04903262) and could possibly provide more definite answers on the use of ECCO_2_R in ARDS [87].

## Pharmacokinetics and antibiotic therapy during VV ECMO

When patients receive ECMO support, important changes in drug pharmacokinetics can occur depending on interactions with the ECMO device, drug characteristics, and the patient’s clinical status [88].

The ECMO circuit itself may behave as an additional compartment by sequestering drugs, increasing volume of distribution (Vd), and changing drug clearance (CL) and elimination; moreover, the circuit may continuously release sequestered drugs even after their administration has stopped [89]. The extent of binding is influenced by drug properties such as molecular weight, plasma protein binding, degree of ionization, and lipophilicity. For instance, drugs with high lipophilicity (such as fentanyl, propofol and voriconazole) [90, 91] and/or high protein binding (such as vancomycin and ceftriaxone) [92] are more likely to be sequestered in ECMO circuits, resulting in a higher loss of the drugs [89]. Materials obviously influence the degree of drug binding, and higher drug concentrations were reported in silicone membrane oxygenators than in hollow-fiber microporous membrane oxygenators; however, there is still disagreement regarding whether the age of a circuit alters drug sequestration [91, 93].

Capillary penetration, fluid shifts and retention, pH, and plasma protein binding of drugs are all mechanisms that may lead to an increased Vd [89]. Besides, renal dysfunction, often present in ECMO patients, causes increased exposure to drugs excreted by the kidney, and continuous renal replacement therapy (CRRT) variably influences drug pharmacokinetics; however, limited data regarding the impact of the combination ECMO-CRRT on the pharmacokinetics of drugs are available [94].

Relevant variability in the pharmacodynamics and kinetics of antibiotics during ECMO has been shown in numerous studies [88, 93]; these differences may be associated with either underdosing (risk of treatment failure) or overdosing (risk of adverse events). Empirical dose adjustment is substantially impossible due to a lack of predictability. Consequently, the best approach towards personalized dosing of antibiotics is the use of therapeutic drug monitoring (TDM) [95]. Although the implementation of a TDM-based antibiotic stewardship program poses clinical, educational, and logistical challenges [96], it has been shown to be clinically beneficial and is recommended by scientific societies [97].

## Weaning from V-V ECMO

The choice of the moment to start the weaning from the extracorporeal support and how to proceed is probably one of the most neglected research areas on ECMO.

The trigger for an ECMO weaning trial usually includes resolution of the underlying disease and patient tolerance of the target ventilator support criteria while maintaining adequate oxygenation and normocarbia [98]. Some experts proposed a multi-step, physiology-based algorithm comprising a reduction in membrane lung FiO_2_ (“ECMO Deoxy Challenge Test”), followed by a progressive reduction of sweep gas flow (“ECMO CO_2_ Challenge Test”), combined with continuous evaluation of gas exchange and inspiratory effort variables [99].

Some observational studies have described the most common reasons for weaning failure from ECMO. Almost invariably, they all showed that the respiratory system and/or lung mechanics are among the most important factors influencing the possibility of weaning. Moreover, markers of increased effort (such as P_0.1_ or esophageal pressure swings) nicely correlated with weaning failure [100]. More importantly, oxygenation variables usually have no correlation with weaning failure, thus underscoring the higher importance of respiratory variables other than gas exchange [101].

## From respiratory support to extracorporeal organ support (ECOS)

In the course of a critical illness, it is common for multiple organ systems to be affected, with the initial impairment of one organ function frequently leading to damage to other organs. Although the etiology, primary affected organ, and underlying mechanisms may differ, the presence of crosstalk between organs ultimately leads to a progressive dysfunction of all organs involved and a significant deterioration in the clinical condition [1, 102].

A standard clinical and pharmacological approach may not provide adequate support for critically ill patients who develop multiple organ dysfunction. Combined lung and kidney failure [103] or liver and kidney failure (hepatorenal syndrome) [104] are typical examples of multiple organ dysfunction in the course of a critical illness, and much of the current knowledge in multiorgan support has been gained from the connection of CRRT to other organ support therapies, such as V-V ECMO [105–107].

Due to the crosstalk between native (and possibly artificial) organs, the effects of multiple extracorporeal techniques may enhance each other (as in kidney support leading to better hemodynamics and circulatory support leading to improved renal function), finally improving patient outcomes [106].

However, as for other very complex treatment options, current knowledge on ECOS is still lacking, especially in terms of precise indications, modes, timing, and duration [105]. The other critical point is the definition of adequate organizational models to deliver ECOS in a safe, appropriate, and cost-efficient manner [1].

## Conclusions and future directions

V-V ECLS techniques have proven to be valuable tools in the management of acute respiratory failure and other critical conditions.

As we look to the future, several areas of research and development stand out.

The indications for V-V ECMO have been established primarily in the context of severe acute respiratory distress syndrome (ARDS) and other forms of acute hypoxemic respiratory failure. However, the gray areas in its use and the absence of absolute contraindications underline the need for careful patient selection and evaluation. On the other hand, ECCO_2_R is still waiting for a definite place in the management of moderate ARDS and acute exacerbations of COPD, and its use should be kept limited to the ongoing research protocols until safety and efficacy are better elucidated.

The quest for smaller, more portable, and more durable ECLS equipment is ongoing, with the goal of increasing patient mobility and reducing the limitations of current systems. Improving oxygenators remains a priority, and microfluidic technology shows promise in providing more efficient and compact oxygenators with lower thrombogenicity.

Coagulation management during V-V ECMO is a critical aspect of patient care, and finding the best anticoagulation regimen and coagulation monitoring systems is essential. However, research and development of less thrombogenic materials and advanced coatings for circuit surfaces may be key to revolutionizing the field and should be prioritized.

The use of protective ventilation during ECMO and how to wean a patient from extracorporeal support, especially in those cases where high inspiratory efforts are recorded, are still matters of debate and should also be placed high on the list of research priorities in the field.

Finally, an integrated approach to multiple organ support is on the rise, with the hope that the synergistic effects of different techniques may further improve the outcomes of critically ill patients and help shape the future of extracorporeal life support.

## Data Availability

The datasets supporting the conclusions of this article are included within the article.

## Abbreviations

ACT: Activated clotting time
AKI: Acute kidney injury
anti-Xa: Anti-factor Xa activity
aPTT: Activated partial thromboplastin time
ARDS: Acute respiratory distress syndrome
AT-III: Antithrombin III
bpm: Breaths per minute
CL: Clearance
CO_2_: Carbon dioxide
COPD: Chronic obstructive pulmonary disease
CRRT: Continuous renal replacement therapy
DTI: Direct thrombin inhibitor
ECCO_2_R: Extracorporeal removal of carbon dioxide
ECLS: Extracorporeal life support
ECMO: Extracorporeal membrane oxygenation
ECOS: Extracorporeal organ support
FiO_2_: Inspiratory fraction of oxygen
FmO_2_: Membrane fraction of oxygen
HIT: Heparin-induced thrombocytopenia
ILAD: Intravascular lung assist device
ML: Membrane lung
NIV: Non-invasive ventilation
PaCO_2_: Arterial partial pressure of carbon dioxide
PaO_2_: Arterial partial pressure of oxygen
PBW: Predicted body weight
PEEP: Positive end-expiratory pressure
PMP: Polymethylpentene
Pplat: Airway plateau pressure
RCT: Randomized controlled trial
RR: Respiratory rate
TDM: Therapeutic drug monitoring
UFH: Unfractioned heparin
V-V: Veno-venous
VCO_2_: Production of carbon dioxide
Vd: Dead space
VILI: Ventilator-induced lung injury
VO_2_: Oxygen uptake
